# High expression of L-GILZ transcript variant 1 (GILZ TV 1) is associated with increased 30-day sepsis mortality, and a high expression ratio possibly contraindicates hydrocortisone administration

**DOI:** 10.1186/s13054-024-05056-1

**Published:** 2024-08-12

**Authors:** Stefan Rusev, Patrick Thon, Birte Dyck, Dominik Ziehe, Tim Rahmel, Britta Marko, Lars Palmowski, Hartmuth Nowak, Björn Ellger, Ulrich Limper, Elke Schwier, Dietrich Henzler, Stefan Felix Ehrentraut, Lars Bergmann, Matthias Unterberg, Michael Adamzik, Björn Koos, Katharina Rump

**Affiliations:** 1 Klinik für Anästhesiologie, Intensivmedizin und Schmerztherapie, Universitätsklinikum Knappschaftskrankenhaus Bochum, 44892 Bochum, Germany; 2 Center for Artificial Intelligence, Medical Informatics and Data Science, University Hospital Knappschaftskrankenhaus Bochum, 44892 Bochum, Germany; 3 Klinik für Anästhesiologie, Intensivmedizin und Schmerztherapie, Klinikum Westfalen, 44309 Dortmund, Germany; 4 Department of Anesthesiology and Operative Intensive Care Medicine, University of Witten/Herdecke, Cologne Merheim Medical School, 51109 Cologne, Germany; 5 Department of Anesthesiology, Surgical Intensive Care, Emergency and Pain Medicine, Ruhr-University Bochum, Klinikum Herford, 32049 Herford, Germany; 6 Klinik für Anästhesiologie und Operative Intensivmedizin, Universitätsklinikum Bonn, 53127 Bonn, Germany

**Keywords:** Sepsis, Mortality, GILZ, Glucocorticoid-induced leucine zipper, TSC22D3, Hydrocortisone, GILZ Transcript variant

## Abstract

**Background:**

Sepsis presents a challenge due to its complex immune responses, where balance between inflammation and anti-inflammation is critical for survival. Glucocorticoid-induced leucine zipper (GILZ) is key protein in achieving this balance, suppressing inflammation and mediating glucocorticoid response. This study aims to investigate GILZ transcript variants in sepsis patients and explore their potential for patient stratification and optimizing glucocorticoid therapy.

**Methods:**

Sepsis patients meeting the criteria outlined in Sepsis-3 were enrolled, and RNA was isolated from whole blood samples. Quantitative mRNA expression of GILZ transcript variants in both sepsis patient samples (n = 121) and the monocytic U937 cell line (n = 3), treated with hydrocortisone and lipopolysaccharides, was assessed using quantitative PCR (qPCR).

**Results:**

Elevated expression of GILZ transcript variant 1 (GILZ TV 1) serves as a marker for heightened 30-day mortality in septic patients. Increased levels of GILZ TV 1 within the initial day of sepsis onset are associated with a 2.2-[95% CI 1.2–4.3] fold rise in mortality, escalating to an 8.5-[95% CI 2.0–36.4] fold increase by day eight. GILZ TV1 expression is enhanced by glucocorticoids in cell culture but remains unaffected by inflammatory stimuli such as LPS. In septic patients, GILZ TV 1 expression increases over the course of sepsis and in response to hydrocortisone treatment. Furthermore, a high expression ratio of transcript variant 1 relative to all GILZ mRNA TVs correlates with a 2.3-fold higher mortality rate in patients receiving hydrocortisone treatment.

**Conclusion:**

High expression of GILZ TV 1 is associated with a higher 30-day sepsis mortality rate. Moreover, a high expression ratio of GILZ TV 1 relative to all GILZ transcript variants is a parameter for identifying patient subgroups in which hydrocortisone may be contraindicated.

**Supplementary Information:**

The online version contains supplementary material available at 10.1186/s13054-024-05056-1.

## Introduction

Sepsis is a multifactorial immunologic syndrome, in which the inflammatory and the anti-inflammatory immune response occur simultaneously and excessively [1]. To achieve higher survival rates, it seems important, that these two reactions remain in a balanced relationship to each other [2]. Both pro-inflammatory and anti-inflammatory overreaction of the immune system, as well as the disproportionate relationship between them, are associated with increased mortality [3]. Therefore, the unbalanced ratio of pro- and anti-inflammation is remaining a main challenge during sepsis [4]. However, it is unknown what ratio is beneficial for each individual patient, how is this ratio changed in specific stages of the sepsis course, such as the early and the late disease phase, as well as the regulatory mechanisms for a balanced immune response [5]. Therefore, it is necessary to investigate the regulatory mechanisms, which are responsible for a fine-tuned and coordinated relationship between pro- and anti-inflammation [6]. The glucocorticoid-induced leucine zipper (GILZ) can potentially act as key protein for attaining a balanced immune response [7]. GILZ is a significant player in the glucocorticoid system [8]. The glucocorticoid system is an important regulatory framework with significant clinical relevance and regulates the pro- and anti-inflammatory response. In addition, GILZ, a TSC22D3 gene product, is identified as a promising protein for sepsis survival in a mouse model [9, 10]. The importance of GILZ in sepsis can be attributed to its ability to suppress inflammation in the innate and adaptive part of the immune system [11, 12]. Furthermore, GILZ takes control over the endogenous glucocorticoid secretion via negative feedback loop mechanism, given that the inflammation process triggers the activation of the glucocorticoid system [13]. Thus, by impacting on the endogenous cortisol synthesis in sepsis, GILZ changes the necessity for treatment with exogenous glucocorticoids. Previous studies have identified GILZ transcript variants (TVs) and their biological activity; nevertheless, the impact of these variants on sepsis survival in humans has not been further investigated [14–16]. For this reason, the intent of the present research was to assess the GILZ transcript profile in a cohort of sepsis patients. In addition, as a potential regulatory mechanism, the expression of the TVs was examined in cell culture after incubation with hydrocortisone, dexamethasone, or lipopolysaccharides (LPS), in order to analyse whether glucocorticoids or an inflammatory trigger can influence the GILZ TV expression. Finally, we examined whether the expression of GILZ TVs can be utilized for the stratification of patients who would benefit from glucocorticoid therapy. Therefore, the main hypotheses of the current study are as follows: 1) the expression of specific GILZ TVs is differentially regulated during sepsis, 2) glucocorticoids and not inflammation upregulate GILZ expression and 3) GILZ mRNA expression alterations can be utilized for identifying patient subgroups with differing sepsis mortality.

## Materials and methods

### Patient recruitment

Sepsis patients were recruited from SepsisDataNet.NRW (NRW-North Rhine-Westphalia, Germany) The NRW project was approved by the Ethics Committee of the Medical Faculty of the Ruhr University of Bochum (No. 18–6606-BR), as well as by the Ethics Committees of the University of Münster (No. 2017–513-b-S) and the University of Bonn. All patients fulfilled criteria according to the Sepsis-3 definition [17]. Patient studies were performed at the hospital Knappschaftskrankenhaus Bochum GmbH (University of Bochum), hospital St. Elisabeth Gruppe GmbH and hospital Herford (University of Bochum), as well as in the clinics for anesthesiology (University of Münster and University of Bonn), following the SepsisDataNet.NRW program (German clinical trial registry: DRKS00018871) [18–20]. The patients were recruited in the period between March 1, 2018, and December 31, 2019 in the ICUs of seven German university clinics. In this project, peripheral blood was obtained on day one, within the first 36 h after sepsis diagnosis, and on day four and on day eight. Characteristics of the these patients can be found in Table 1. In addition, a postoperative control cohort was included, which consisted of post-operative patients, which did not develop sepsis in the post-operative course. Characteristics of the control patients can be found in Table 2. Written informed consent was given by all patients or their legal representatives.

**Table 1:** Baseline characteristics of sepsis patients

	Total cohort
Sex male n (%)	70 (57.9%)
Age in years median	67 (59–79)
SOFA Score median—Day 1	8 (6–12)
30-day mortality (%)	54 (44.6%)
Hydrocortisone treatment	32 (26.4%)
CRP – Day 1 median	14.33 (9.27–24.69)
PCT – Day 1 median	4.63 (0.59–14.1)
ICU length of stay (days) median	6.77 (2.96–14)
Infection focus n (%)	
CNS	2 (1.65%)
Lower respiratory tract	46 (38%)
Skin and soft tissues	7 (5.7%)
Urogenital tract	10 (8.2%)
Cardiovascular	8 (6.6%)
Intraabdominal	24 (19.8%)
Musculoskeletal	3 (2.4%)
Unknown	21 (17.3%)

**Table 2:** Baseline characteristics of control patients

	Total cohort
Sex male n (%)	38 (52.1%)
Age in years median	64 (56.5–76)
SOFA Score median—Day 1	3 (1–6)
30-day mortality (%)	0 (0%)
Infection n (%)	0 (0%)
Comorbidities n (%)	
Alkohol	8 (11.0%)
Nicotine	26 (35.6%)
Lung/COPD	11 (15.1%)
Hypertension	46 (63.0%)
Chronic kidney disease	4 (5.5%)
Diabetes	9 (12.3%)
Obesity	15 (20.5%)
Cardio-vascular	23 (31.5%)
Malignant	58 (79.5%)
Dialysis	0 (0%)
Other	1 (1.4%)

### Blood sample collection, preparation, and storage

Whole blood was taken from patients immediately processed [17]. For immediate isolation of RNA, an RNA Blood Tube (Applied Biosystems, Life Technologies, Darmstadt, Germany) was used obtained from septic patients on day one and eight. Not all patients had samples available at both time points (Supplementary Table 5). RNA was extracted from whole blood samples with a Tempus™ Spin RNA (Applied Biosystems) kit in accordance with the manufacturer’s instructions. A high-capacity cDNA reverse transcription kit (Applied Biosystems) was used for reverse transcription of the messenger RNA (mRNA) into complementary DNA (cDNA). RNA sample aliquots were stored at −80 °C.

### GILZ-mRNA quantification

The gene expression analysis was performed with quantitative polymerase chain reaction (qPCR) in duplicate using GoTaq® qPCR Master Mix (Promega, Madison, WI, USA) or ZymoTaq qPCR Premix (Zymo Research, Irvine, CA, USA) for the related primers (Eurofins Scientific SE, Hamburg, Germany) (Supplementary Table 1) on a CFX Connect Real-Time System (Bio-Rad Laboratories, Hercules, CA, USA). For GILZ (TSC22D3) TVs No. 1–5, the corresponding GoTaq® qPCR Master Mix or ZymoTaq qPCR Premix was used (Supplementary Table 1). A total of 25 µL of reaction mixture (containing 12.5 µL of GoTaq qPCR Master Mix or ZymoTaq qPCR Premix, 2.5 µL of cDNA (10 ng/µL), 1 µL of forward primer, 1 µL of reverse primer, and 8 µL of nuclease-free H₂O (Promega, Madison, WI, USA)) was added to a 96-well reaction plate (Sarstedt, Nümbrecht, Germany). q-PCR amplification was completed using the following protocols: GoTaq qPCR® Master Mix—a denaturation cycle at 95 °C for 2 min, followed by 40 cycles of 95 °C for 30 s and the respective annealing temperature for 30; ZymoTaq qPCR Premix—a denaturation cycle at 95 °C for 10 min, followed by 40 cycles of 95 °C for 30 s, accompanied by the corresponding annealing temperature for 30 s and an extension step at 72 °C, with a final extension at 72 °C for 7 min. The relative GILZ-mRNA expression was adjusted to the expression of the reference gene β-actin (Integraed DNA Technologies, Coralvillem, IA, USA) and, if not otherwise mentioned, was computed using the 2^−∆CT^ method.

### Quantification of cytokines in serum

Serum samples collected upon admission were analyzed for thirteen cytokines. The LEGENDPlex™ Human Inflammation Panel 1 (BioLegend, San Diego, CA, USA) was employed following the manufacturer’s protocol. Briefly, 25 µL of serum was incubated with LEGENDPlex™ beads for antigen capture, followed by washing and incubation with detection antibodies. Fluorescence intensity was then measured using a flow cytometer (Canto II, BD Biosciences, San Jose, CA, USA). Cytokine concentrations falling below the lower limit of detection (LOD) were recorded as 0 pg/mL, while concentrations exceeding the upper LOD were recorded as the LOD value itself.

### Cell culture experiments with U937 cells

We used the U937 cell line to test the effect of hydrocortisone and LPS on TSC22D3 transcript variant expression. The U937 cells were purchased from DSMZ (Leipzig, Germany). The cells were cultured in RPMI 1640 medium (PAN-Biotech, Aidenbach, Germany) supplemented with heat-inactivated 10% FCS (PAN-Biotech)), penicillin (100 U/ml) and streptomycin (100 μg/ml) (both Thermo Fisher Scientific, Bremerhaven, Germany). The cells were maintained in a humidified atmosphere at 37 °C with 5% CO_2_ and were split three times a week to maintain a cell density of approximately 10^6^ cells/ml. Cells from early passages (< 25) were used for all tests, seeded in 6-well plates and stimulated with 10^−6^ M hydrocortisone, 10^−6^ M dexamethasone, 10 µg/ml LPS or 100 ng/ml LPS (all from Sigma‒Aldrich, Taufkirchen, Germany) or left unstimulated (control).

Cell stimulation was stopped after 2 h, 6 h or 24 h of cell lysis, and RNA was isolated with an RNeasy Mini Kit (Qiagen, Hilden, Germany). The purified mRNA samples were reverse transcribed into complementary DNA using a high-capacity cDNA reverse transcription kit (Applied Biosystems).

### Statistical analyses

The characteristics of the recruited patients are given as numbers and percentages for categorical variables, means and standard deviations (± SD), or medians with corresponding interquartile ranges for continuous variables. After generating the ROC curve and computing the Youden index, a cutoff value was determined for each of the presented TSC22D3 transcript variants and for the computed ratio (ΔCt GILZ TV 1 relative to all transcript variants) as the point of best discrimination between sepsis survivors and non-survivors. The survival rate of the septic patients, divided by the cutoff value for the corresponding TSC22D3 transcript variant or TV 1 expression ratio, was estimated by Kaplan‒Meier survival curves. To determine the statistical significance of the observed survival effect, a log rank test was performed. Hazard ratios with corresponding 95% confidence intervals (HRs) were determined using univariate Cox regression analysis or multivariate Cox regression to estimate the independence of each TSC22D3-mRNA transcript variant on survival and to assess the associations between covariates and mortality. All assessed variables were tested for a normal distribution with the Shapiro‒Wilk test. For comparisons of continuous variables, an unpaired t test, a Mann‒Whitney-U test or one-way ANOVA was used. P values lower than 0.05 were considered to indicate statistical significance. All the statistical calculations were performed with SPSS Statistics (Version 28.0.0.0, IBM, Armonk, NY, USA), and the graphics were created with GraphPad Prism 8 (Graph-Pad, San Diego, CA, USA).

## Results

### Patient characteristics

Samples from 121 patients diagnosed with sepsis were analyzed. Most septic patients were male (57.9%), the median sequential organ failure assessment (SOFA) score was 8, the 30-day mortality was 44.6%, and the main focus of infection was the lower respiratory tract (Table 1).

### GILZ transcript variant expression in patients during sepsis

**Fig. 1:**
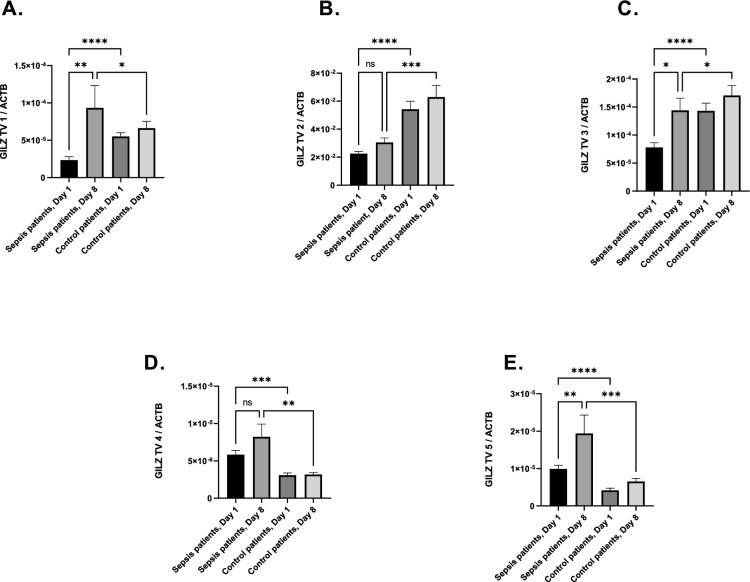
Quantitative GILZ-mRNA expression of TV 1–5 in patients one and eight days after sepsis diagnosis. The expression was normalized to that of the housekeeping gene ß-actin (ACTB) using the 2 − ∆CT formula. day one, n = 105; day eight, n = 53; *p = 0.0121; **p = 0.004; ***p = 0.0007; ****p < 0.0001

In a first step, we examined whether GILZ TV expression could be relevant in sepsis. We measured the expression of all GILZ TV on days one and eight after sepsis diagnosis (Fig. 1A–E). GILZ mRNA expression increased in transcript variants 1,3, and 5 from day one to day eight. The expression of GILZ TV 1 (p = 0.0072, Fig. 1A) increased nearly fivefold, and the expression of GILZ TV 5 more than doubled (p = 0.0014; Fig. 1E). No significant increase could be seen in transcript variant 2 and 4 (Fig. 1B, D). In the postoperative control cohort GILZ expression differed from septic patients. GILZ mRNA level seemed to be reduced in septic patients in transcript variants 1, 2, 3, where it was increased at the onset of sepsis in transcript variants 4 and 5 (Fig. 1A–E). In a next step we examined the impact of GILZ transcript variant expression on sepsis survival. After plotting the receiver operating characteristic curve and calculating the area under the curve (AUC) for each GILZ TV (Supplementary Table 2), we focused our investigations on GILZ TV 1: The classification threshold models for this TV had the best performance in survival analysis (AUC: 0.628 for day 1 and AUC: 0.783 for day 8) with a p value lower than 0.05. The selected threshold values were for GILZ TV 1, Day 1—HR = 2.2, (CI:1.1,4.2), p = 0.016, Cut-off = 2.543*10^−6^; and for GILZ TV 1, Day 8—HR = 8.5, (CI:1,9,36,38), p = 0,004, Cut-off = 7.604*10^−6^.

### Survival analysis of sepsis patients expressing GILZ transcript variant 1 on days one and eight after sepsis onset

**Fig. 2:**
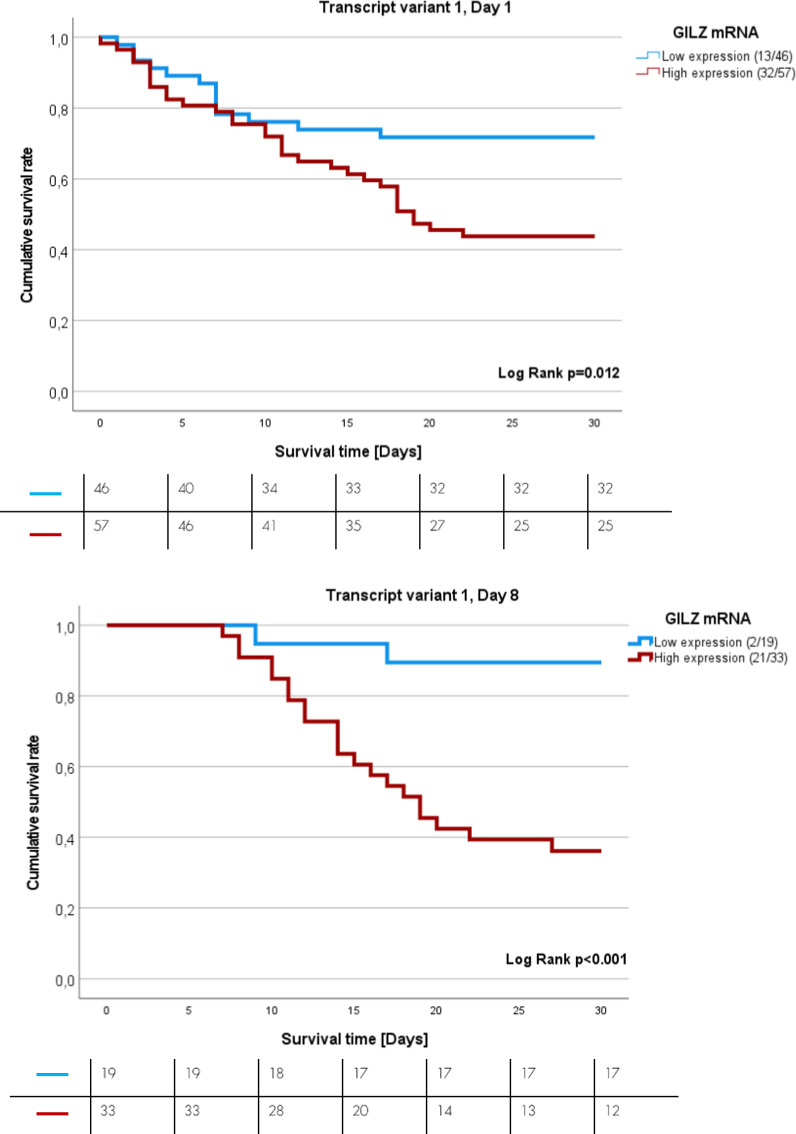
K-M curves showing the 30-day survival rate of sepsis patients. **A** Patients with low (blue) or high (red) GILZ-mRNA TV 1 expression on the day of sepsis onset (HR = 2.2, p = 0.016, 95% CI 1.1–4.2). **B** Patients with low (blue) or high (red) GILZ-mRNA TV 1 expression on day eight after sepsis onset (HR = 8.5, p = 0.004, 95% CI 1.98–36.28). Numbers in brackets represent the number of dead patients in relation to total tested patient number

After performing ROC analysis and computing the Youden index for identifying the best point of discrimination between sepsis survivors and non-survivors, Kaplan–Meier (K-M) analysis was performed. K-M curves revealed the impact of GILZ TV1 on the 30-day survival rate (Fig. 2A‒B). High mRNA expression of GILZ TV 1 on day one and day eight (red lines) is associated with increased sepsis mortality. There was a 2.2-fold increase in sepsis mortality in patients with high levels of GILZ TV 1 on day one (p = 0.015; 95% CI 1.1–4.2, Cut-off = 2.543*10^−6^, Fig. 2A), and there was an 8.5-fold increase in sepsis mortality in those with high levels of GILZ TV 1 on day eight (p = 0.004; 95% CI 1.98–36.28, Cut-off = 7.604*10^−6^, Fig. 2B).

High expression of GILZ TV 1 on day one was a risk factor for 30-day mortality (HR = 2.183; 95% CI (1.16–4.01); p = 0.015) and patients did not differ in SOFA-score or other characteristics (Supplementary Table 3). In a multivariate Cox regression high GILZ TV 1 expression on day one was no longer an independent risk factor (Supplementary Table 4). On the other side, high GILZ TV 1 expression on day eight was even a stronger risk factor for sepsis mortality (HR = 10.569, p = 0.027; 95% CI 1.309–85.309), independent of SOFA score on day, age, sex and hydrocortisone treatment according to the multivariate Cox regression model (Table 3).

**Table 3:** Univariate and multivariate cox regression for GILZ TV 1, day eight

	Univariate				Multivariate			
	HR	p-value	95.0% confidence intervals for HR		HR	p-value	95.0% confidence intervals for HR	
			Lower	Upper			Lower	Upper
GILZ Transcript variant 1, Day 8	8.750	0.003	2.050	37.350	7.753	0.007*	1.729	34.760
SOFA-Score Day 1	1.276	> 0.001	1.185	1.373	1.095	0.237	0.942	1.273
Age	1.007	0.422	0.989	1.026	1.011	0.544	0.975	1.049
Sex	0.993	0.981	0.579	1.704	1.719	0.294	0.625	4.728

### Impact of hydrocortisone and LPS on GILZ transcript variant expression in the U937 cell line

**Fig. 3:**
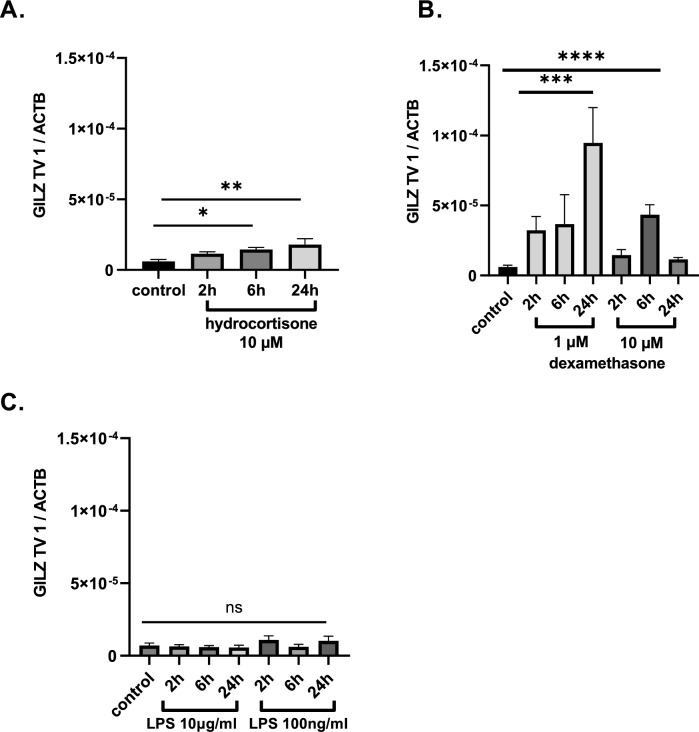
Quantitative GILZ-mRNA expression of TV 1 in U937 cells after incubation with **A** hydrocortisone (10 µM) or **B** dexamethasone (1 µM and 10 µM) and **C** LPS (10 µg/ml and 100 ng/Ml] The expression was normalized to that of the housekeeping gene ß-actin using the 2 − ∆Ct formula (**A** *p = 0.0309, **p = 0.003, n = 3; **B** ***p = 0.0003, n = 3; **C** p = n.s., n = 3)

In a second step, we investigated possible regulatory mechanisms for GILZ-mRNA expression in septic patients. We stimulated U937 cells with hydrocortisone (10^−6^ M), dexamethasone (10^−6^ M) and LPS (10 µg/ml and 100 ng/ml) for 2 h, 6 h and 24 h. Compared to the control sample, incubation with hydrocortisone for 6 h and 24 h resulted in higher expression of GILZ TV 1 (*p = 0.0309, **p = 0.003, n = 3; Fig. 3A -). Further, incubation for 24h with dexamethasone led also to higher GILZ TV 1 concentration (- ***p = 0.0003, n = 3; Fig. 3B). LPS stimulation did not affect GILZ TV 1 expression at all (p = n.s., n = 3; Fig. 3C).

### Impact of hydrocortisone on GILZ transcript variant expression and the survival of septic patients

**Fig. 4:**
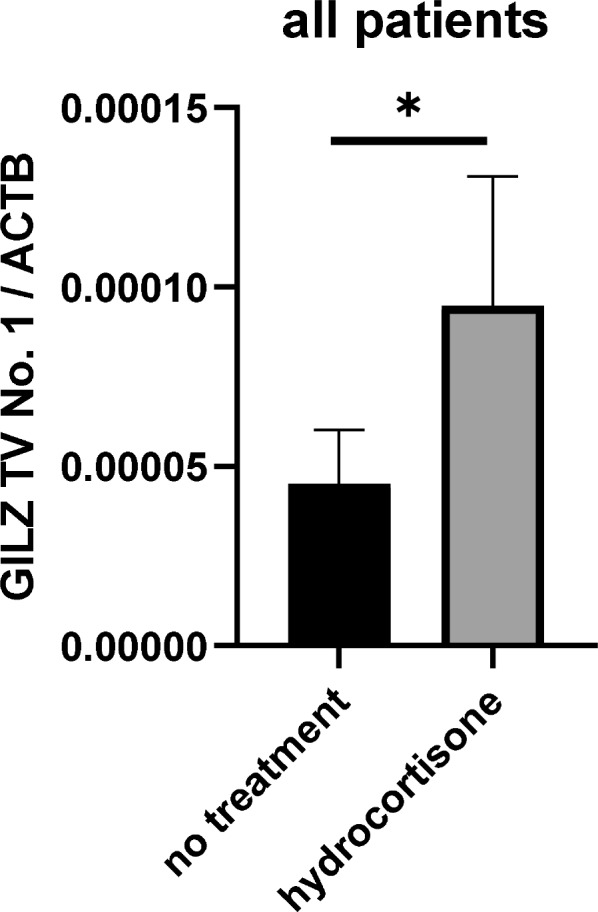
Effect of hydrocortisone on the concentration of GILZ TV 1 on day eight, *p = 0.023 no treatment n = 29, hydrocortisone n = 13

Furthermore, we wanted to confirm whether the effect of hydrocortisone on GILZ TV 1 can also be observed in septic patients. We noticed that, if hydrocortisone was given prior day eight of sepsis onset, then patients had higher expression levels of GILZ TV 1 on day 8 (Fig. 4, p = 0.023).

**Fig. 5:**
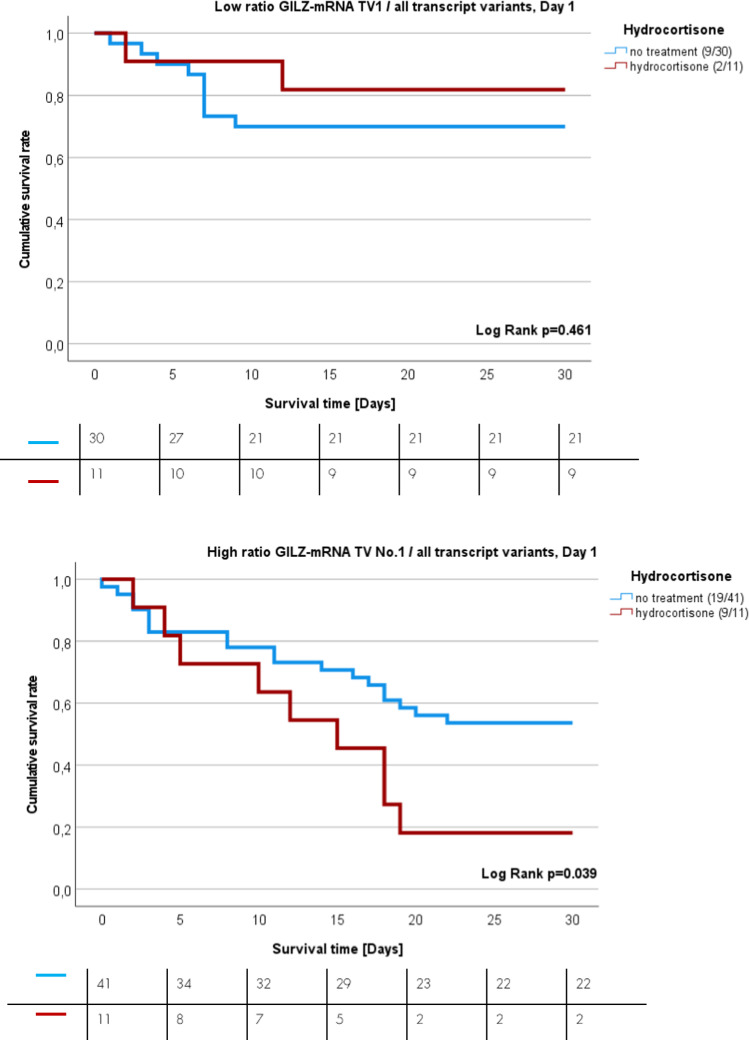
K-M curves showing the 30-day survival rate of sepsis patients treated with hydrocortisone. **A** HR = 0.549, p = 0.443 95% 0.11–2.54. **B** HR = 2.38, p = 0.035, 95% CI 1.23–4.62. Numbers in brackets represent the number of dead patients in relation to total tested patient number

Finally, we investigated whether hydrocortisone treatment affects sepsis survival in a manner dependent on GILZ-TV1 expression. Patients were stratified into two main groups: a group with low expression ratio of GILZ TV 1 relative to all transcript variants (Fig. 5A) and a group with high expression ratio (Fig. 5B). The calculated ratio of GILZ TV 1 relative to all other GILZ TVs was calculated based on the expression values on day one for GILZ TV 1 and all other GILZ TVs. Then, each group was separated into no corticosteroid treatment or hydrocortisone treatment sub-groups. Patients with low GILZ TV 1 expression quotient, below the cut-off, had similar 30-day sepsis mortality rate, regardless of glucocorticoid treatment (blue line) or no hydrocortisone treatment at all (red line) (Fig. 5A, HR = 0.549, p = 0.443, 95% CI 0.11–2.54). If the expression ratio between GILZ TV 1 relative to all transcript variants surpasses the threshold, indicating a high expression quotient, then patients who received hydrocortisone treatment (red line) had 2.38-fold greater sepsis mortality rate than patients who had high expression ratio and did not receive glucocorticoid therapy at all (Fig. 5B, p = 0.035). In multivariate Cox regression models the GILZ TV 1 expression ratio was an independent risk factor for survival independent of hydrocortisone treatment (HR = 2.303, p = 0.020; 95% CI 1.139–4.658) and septic shock (HR = 2.333, p = 0.022; 95% CI 1.129–4.820) (Supplementary Tables 6A-B). However, this effect was not independent of the SOFA score (HR = 2.1, p = 0.106, 95% CI 1.23–4.62).

## Discussion

Our study demonstrates that GILZ TV 1 is associated with the 30-day mortality rate in sepsis patients. Increased expression of GILZ TV 1 seems to come along with a higher sepsis mortality rate. High expression of GILZ TV 1 on day eight is an independent risk factor for mortality, with a hazard ratio of 10.569, independent of well-known risk factors such as SOFA score, age and sex and of hydrocortisone administration in a multivariate COX regression. The expression of GILZ TV 1 seems to be upregulated via exogenous and endogenous glucocorticoids but nor by inflammatory stimulation with LPS. Additionally, in patients exhibiting a high ratio of GILZ TV1 to other transcript variants, supplemental administration of hydrocortisone appears to exacerbate the outcome further.

First, we investigated the expression of which GILZ transcript variant can be associated with sepsis survival. We discovered that GILZ TV 1 appeared to be related with sepsis survival rate, in a such way, that high GILZ TV 1 expression was in a multivariate Cox regression a dependent risk factor for mortality on day 1 with HR = 1.318 and independent risk factor for mortality on day 8 with HR = 10.569. One could speculate that patients with high GILZ TV 1 expression on day 8 might be prone for late complications such as nosocomial infections, since high expression of GILZ TV 1 on day 8 is an independent risk factor for mortality, independent of SOFA Score, age, sex and hydrocortisone treatment. In subsequent research focused solely on this transcript variant GILZ TV 1. In this context, we can speculate about the precise molecular mechanisms how and why GILZ TV 1 can be associated with the mortality. GILZ TV 1 encodes for GILZ protein isoform 1 (GILZ1) [21].

In the following paragraph, we would like to make some speculations about GILZ TV 1 mediated mechanisms. GILZ TV 1 is the longest of all GILZ isoforms and possesses an extensive N-terminus [22]. Hence the extended N-terminus of GILZ 1 could possibly be responsible for specific target binding of this isoform,[23]. The GILZ N-terminal domain is responsible for interaction with activator protein 1 (AP1) and Raf (rapidly accelerated fibrosarcoma) proteins [16, 24]. Therefore, GILZ TV1 could bind AP1 and Raf and blockade the associated pathways [25, 26], which can lead to the induction of proinflammatory cytokines [27–29]. In our study we found a negative correlation of GILZ TV 1 ration with IL-18, IL-23 and to a weaker extent to MCP-1 and IL-6 (Supplementary Table 7). In this regard one could speculate that inhibition of AP1, as well as Raf via the N-terminus of GILZ TV 1 might restrain the pro-inflammation significantly, further it could shift the immune balance towards anti-inflammation and it could support secondary infections [30, 31]. In this context, high expression of GILZ TV1 may contribute to lower 30-day sepsis survival rates, potentially due to its association with immune suppression which increase susceptibility to secondary infections and exacerbate mortality [32–35]. This assumption might be confirmed by the fact that especially the GILZ expression in the late phase (day 8) of sepsis seems to be relevant.

In a second step, we tried to pinpoint the mechanisms of high GILZ TV 1 expression. LPS as a pathogen-associated molecular pattern (PAMP) showed no impact on GILZ TV1 expression. Hence, the inflammation signal LPS is no regulator of GILZ TV1 expression in the tested time frame and cell line. Our observations underline that glucocorticoids remain the key inductors of GILZ. Dexamethasone and hydrocortisone led to higher GILZ TV1 expression, whereby the effect of dexamethasone was around 3-times higher than the effect of hydrocortisone. This cell culture experiments showed that GILZ expression is increased by exogenous glucocorticoids.

Besides, we could see that the quantitative expression of GILZ transcript variants was significantly increased in the time course of sepsis. As our cell culture experiments indicate no upregulation by LPS, this seems not to be caused by the infection itself. As only 26.4% of these sepsis patients were prescribed exogenous glucocorticoids (hydrocortisone), the heightened expression in the remaining 73.6% might be attributed to endogenous cortisol. This is a crucial finding, since it indicates, that endogenous glucocorticoids do play a significant role in the observed increased expression of GILZ TV 1. Endogenous glucocorticoids are synthetised and released after activation of the hypothalamic–pituitary–adrenal (HPA) axis [36]. Hence, the activation of this stress axis seems to be an endogenous regulator for GILZ TV 1 expression. Additionally, hydrocortisone is the preferred glucocorticoid in medical guidelines for the treatment of septic shock [37]. In this context, the prescription of hydrocortisone serves as an exogenous regulator for the expression of GILZ TV 1. Given our demonstration that high expression of GILZ TV 1 is associated with increased sepsis mortality, this raises concerns regarding the potential contraindication of hydrocortisone in patients with elevated GILZ TV 1 expression. Since hydrocortisone, as a glucocorticoid derivative, can impact all other GILZ TVs, an expression quotient between GILZ TV 1 and all other GILZ transcript variants (2–5) was calculated. The expression ratio cut-off value, derived for discriminating between sepsis survivors and non-survivors, was used to categorize patients into low and high GILZ TV 1 groups, with further subgrouping based on hydrocortisone prescription. Importantly, in the group with relatively high GILZ TV 1, hydrocortisone treatment was associated with 2.3-fold higher 30-day sepsis mortality rate than the sub-group with relatively high GILZ TV 1 and no hydrocortisone prescription at all. In this case, relatively high GILZ TV 1 expression quotient could serve as an indicator suggesting that patients should not be prescribed hydrocortisone treatment due to increased mortality risk. Our findings suggest that the expression ratio of GILZ TV 1 relative to other TVs could serve as a novel biomarker, indicating increased 30-day mortality risk with hydrocortisone treatment in patients exhibiting a high expression quotient. Previous research supports that hydrocortisone treatment may not be suitable for specific subgroups of sepsis patients [38–40].

Limitations of our study must be mentioned. Firstly, we have demonstrated in a cell culture model that upregulation of GILZ TV 1 is induced by hydrocortisone and dexamethasone. However, while we observed higher GILZ TV 1 expression levels associated with hydrocortisone prescription prior to day eight, this effect must be validated against a placebo in human studies. Furthermore, we associated a 2.3-fold increase in mortality among patients with relatively high GILZ TV 1 expression who underwent hydrocortisone therapy. However, it remains unclear whether the observed effects of high GILZ TV 1 expression are causative for higher mortality or only associations. Since high GILZ TV 1 expression on day 8 is an independent risk factor for mortality and high GILZ TV 1 ratio is also a risk factor, independent of SOFA Score, hydrocortisone or septic shock, one can speculate, that the mortality effect is probably not only due to the fact, that patients with hydrocortisone treatment are sicker, because they have septic shock and thus prone to higher mortality. It remains also unclear whether patients with relatively low GILZ TV 1 expression would benefit from glucocorticoid therapy for improved survival rates. Thus, the question arises as to whether there exists a therapeutic window for hydrocortisone treatment, wherein it should only be prescribed when the expression quotient of GILZ TV 1 falls within a specific reference range. While we noticed a trend in a specific sub-group of sepsis patients, indicating slightly higher survival rates among those with relatively low GILZ TV 1 expression, further investigation with a larger patient cohort is necessary to validate this observation. Prospective randomized studies are warranted to evaluate whether a therapeutic window exists for hydrocortisone treatment and whether the mortality impact of high GILZ TV 1 expression quotient can be mitigated with a placebo. Additionally, one must evaluate, if sepsis patients would profit only from short duration of a glucocorticoid therapy [41]. In this regard, first attempts have been started [42].

## Conclusion

To summarize, the high expression of GILZ TV 1 depicts a possible biomarker for predicting the mortality rate and the responsiveness of hydrocortisone treatment in sepsis patients. It was illustrated, that high expression of GILZ TV 1 is associated with a higher 30-day mortality rate in sepsis patients and the expression seems to be regulated via exogenous, as well as endogenous glucocorticoids. Furthermore, in patients with a high expression ratio of GILZ TV 1 relative to all GILZ transcript variants, hydrocortisone treatment is associated with a 2.3-fold greater 30-day mortality rate. In this context, prospective randomized studies must assess, if the expression quotient for GILZ TV 1 can establish a therapeutic window regarding hydrocortisone prescription.

### Supplementary Information

Supplementary Materials

## Data Availability

The data that support the findings of this study are not openly available due to reasons of sensitivity and are available from the corresponding author upon reasonable request. Data are located in controlled access data storage at Ruhr University Bochum.
