# Supplementary material for: High expression of L-GILZ transcript variant 1 (GILZ TV 1) is associated with increased 30-day sepsis mortality, and a high expression ratio possibly contraindicates hydrocortisone administration
Source: Crit Care. 2024 Aug 12;28:270. doi: 10.1186/s13054-024-05056-1 (PMC11321204; doi:10.1186/s13054-024-05056-1)
Supplement: Supplementary file 1 — Supplementary Materials [file 13054_2024_5056_MOESM1_ESM.docx]

Supplementary files:

**Supplementary Table 1:** Primers utilized for TSC22D3 transcript variant quantification

|  | **Forward primer sequence 5'->3'** | **Reverse primer sequence 5'->3'** | **Annealing T** | **MasterMix** |
| --- | --- | --- | --- | --- |
| TSC22D3 transcript variant No.1 | TCACACTTGGGAAACTTGGGA | GATGCCCTCGTTGATCAGGT | 60° | Zymo  Taq |
| TSC22D3 transcript variant No.2 | GTTAAGCTGGACAACAGTGCCT | TTCTCCACCAGCTCTCGGAT | 60° | GoTaq |
| TSC22D3 transcript variant No.3 | TAGCTAGCTTCAGAGCCGGTG | AACAGGGTGTTCTCACGCTC | 60° | Zymo  Taq |
| TSC22D3 transcript variant No.4 | CTAGCTCACTCGCTCTCAGC | CCTGGAAAAGACAAGCTGTGG | 63° | Zymo  Taq |
| TSC22D3 transcript variant No.5 | CTAGCTCACTCGCTCTCAGC | GACTCCAGGGTGCCTGTG | 60° | Zymo  Taq |
| TSC22D3 transcript variant No.1-5 | TCCTGTCTGAGCCCTGAAGAG | AGCCACTTACACCGCAGAAC | 60° | GoTaq |
| Β-Actin | CCTTCCTGGGCATGGAGT | CAGGGCAGTGATCTCCTTCT | 60° | GoTaq |

**Supplementary Table 2.** Area under the curve (AUC) values for each GILZ transcript variant on days one and eight after sepsis diagnosis according to receiver operating characteristics.

| **TSC22D3 transcript variant (TV)** | **AUC** | **Asymptotic Significance** | **Asymptotic 95% Confidence Interval** | |
| --- | --- | --- | --- | --- |
|  |  |  | **Lower Bound** | **Upper Bound** |
| TV1, day 1 | 0.628 | *0.030** | 0.515 | 0.736 |
| TV1, day 8 | 0.783 | *0.001^*^* | 0.656 | 0.909 |
| TV2, day 1 | 0.504 | 0.987 | 0.383 | 0.619 |
| TV2, day 8 | 0.601 | 0.214 | 0.445 | 0.757 |
| TV3, day 1 | 0.484 | 0.242 | 0.320 | 0.544 |
| TV3, day 8 | 0.620 | 0.423 | 0.276 | 0.594 |
| TV4, day 1 | 0.462 | 0.527 | 0.340 | 0.583 |
| TV4, day 8 | 0.600 | 0.253 | 0.432 | 0.768 |
| TV5, day 1 | 0.443 | 0.303 | 0.326 | 0.555 |
| TV5, day 8 | 0.642 | 0.082 | 0.491 | 0.792 |

**Supplementary Table 3:** Baseline characteristics of patients stratified by GILZ TV 1 cut-off on day 1

|  | **Below Cut-off (day 1)** | **Above Cut-off (day 1)** | **p-Value** |
| --- | --- | --- | --- |
| Sex male n (%) | 26 (54.2 %) | 33 (58.9 5) | 0.625 |
| Age in years median | 63 (57.5 – 75) | 69 (62- 75) | 0.215 |
| SOFA Score median - Day 1 | 8 (5-12) | 10 (6-11) | 0.333 |
| 30-day mortality (%) | 14 (29.2 %) | 32 (56.1 %) | *0.006* |
| Hydrocortisone treatment | 13 (28.9 %) | 10 (19.6 %) | 0.288 |
| CRP – Day 1 median | 15.8 (8.9 -24.2) | 16.3 (9.7-24.6) | 0.215 |
| PCT – Day 1 median | 3.9 (0.3-14.4) | 5.6 (0.4 – 14.4) | 0.623 |
| ICU length of stay (days) median | 5 (2- 7) | 7 (3-17) | 0.471 |

**Supplementary Table 4.** Multivariate Cox regression for GILZ TV 1 expression on day one.

|  | **Univariate** | | | | **Multivariate** | | | |  |
| --- | --- | --- | --- | --- | --- | --- | --- | --- | --- |
|  | HR | p-value | 95.0% confidence intervals for HR | | HR | p-value | 95.0% confidence intervals for HR | | |
|  |  |  | Lower | upper |  |  | Lower | Upper | |
| **GILZ Transcript variant 1, Day 1** | 2.269 | 0.013 | 1.189 | 4.327 | 1.318 | 0.462 | 0.631 | 2.754 | |
| **SOFA-Score Day 1** | 1.276 | <0.001 | 1.185 | 1.373 | 1.242 | <0.001 | 1.137 | 1.370 | |
| **Age** | 1.007 | 0.422 | 0.989 | 1.026 | 1.015 | 0.221 | 0.991 | 1.041 | |
| **Sex** | 0.993 | 0.981 | 0.579 | 1.704 | 1.014 | 0.968 | 0.503 | 2.044 | |
| **Hydrocortisone** | 1.462 | 0.226 | 0.790 | 2.704 | 1.239 | 0.581 | 0.578 | 2.655 | |

**Supplementary Table 5:** Patients stratified by threshold and data availability at the different time points.

|  | **Total** | **Available at Day 8** | **Only available Day 8** |
| --- | --- | --- | --- |
| **Low expression group Day 1** | 48 | 14 | 3 |
| **High expression group Day 1** | 57 | 27 | 9 |

|  | **Univariate** | | | | **Multivariate** | | | |  |
| --- | --- | --- | --- | --- | --- | --- | --- | --- | --- |
|  | HR | p-value | 95.0% confidence intervals for HR | | HR | p-value | 95.0% confidence intervals for HR | | |
|  |  |  | Lower | upper |  |  | Lower | Upper | |
| **GILZ TV 1 relative to all other transcript variants** | 2.385 | 0.010 | 1.231 | 4.623 | 2.303 | 0.020 | 1.139 | 4.658 | |
| **Hydrocortisone** | 1.462 | 0.226 | 0.790 | 2.704 | 1.455 | 0.297 | 0.719 | 2.943 | |

**Supplementary Table 6A.** Multivariate Cox regression for the expression ratio of GILZ TV 1 relative to

all other GILZ TVs.

|  | **Univariate** | | | | **Multivariate** | | | |  |
| --- | --- | --- | --- | --- | --- | --- | --- | --- | --- |
|  | HR | p-value | 95.0% confidence intervals for HR | | HR | p-value | 95.0% confidence intervals for HR | | |
|  |  |  | Lower | upper |  |  | Lower | Upper | |
| **GILZ TV 1 relative to all other transcript variants** | 2.385 | 0.010 | 1.231 | 4.623 | 2.333 | 0.022 | 1.129 | 4.820 | |
| **Septic Shock** | 5.577 | <0.001 | 2.909 | 10.693 | 5.543 | <0.001 | 2.797 | 10.986 | |

**Supplementary Table 6B.** Multivariate Cox regression for the expression ratio of GILZ TV 1 relative to

all other GILZ TVs.

**Supplementary Table 7:** correlation of GILZ TV 1 ratio to different cytokines in septic patients

|  | | **GILZ TV 1 ratio day 1** |  | | **GILZ TV 1 ratio**  **day 1** |
| --- | --- | --- | --- | --- | --- |
| **TNFa_day 4** | Pearson-Correlation | 0,010 | **TNFa_day 8** | Pearson-Correlation | ,261* |
|  | Sig. (1-tailed) | 0,467 |  | Sig. (1-tailed) | 0,050 |
|  | N | 72 |  | N | 41 |
| **IL1B_** **day 4** | Pearson-Correlation | -0,001 | **IL1B_** **day 8** | Pearson-Correlation | 0,058 |
|  | Sig. (1-tailed) | 0,498 |  | Sig. (1-tailed) | 0,358 |
|  | N | 72 |  | N | 41 |
| **MCP1_** **day 4** | Pearson-Correlation | -,238* | **MCP1_** **day 8** | Pearson-Correlation | 0,142 |
|  | Sig. (1-tailed) | 0,022 |  | Sig. (1-tailed) | 0,187 |
|  | N | 72 |  | N | 41 |
| **IL6_** **day 42** | Pearson-Correlation | -,196* | **IL6_** **day 83** | Pearson-Correlation | -0,092 |
|  | Sig. (1-tailed) | 0,049 |  | Sig. (1-tailed) | 0,283 |
|  | N | 72 |  | N | 41 |
| **IL10_** **day 4** | Pearson-Correlation | -0,083 | **IL10_** **day 8** | Pearson-Correlation | -0,108 |
|  | Sig. (1-tailed) | 0,245 |  | Sig. (1-tailed) | 0,252 |
|  | N | 72 |  | N | 41 |
| **IL18_** **day 42** | Pearson-Correlation | -,435** | **IL18_** **day 8** | Pearson-Correlation | 0,057 |
|  | Sig. (1-tailed) | 0,000 |  | Sig. (1-tailed) | 0,361 |
|  | N | 72 |  | N | 41 |
| **IL23_** **day 4** | Pearson-Correlation | -,296** | **IL23_** **day 8** | Pearson-Correlation | -0,001 |
|  | Sig. (1-tailed) | 0,006 |  | Sig. (1-tailed) | 0,498 |
|  | N | 72 |  | N | 41 |

**Supplementary Figure S1.** Effect of hydrocortisone treatment on the delta value – GILZ TV 1 expression on day eight minus expression on day one.

**Supplementary Figure S2:** Effect of hydrocortisone on the concentration of GILZ TV 1 on day eight, stratified by SOFA-Score. Patients were divided into two groups by their SOFA-Score, (SOFA-Score < 8 n= 17; SOFA-Score > 8 n= 21 *p=0.023, p=0.0043)
